# Supplementary material for: MAPK-mediated transcription factor GATAd contributes to Cry1Ac resistance in diamondback moth by reducing PxmALP expression
Source: PLoS Genet. 2022 Feb 3;18(2):e1010037. doi: 10.1371/journal.pgen.1010037 (PMC8846524; doi:10.1371/journal.pgen.1010037)
Supplement: S1 Fig — The nucleotides are numbered relative to the translation start site (ATG) indicated as +1 and in red font. The position also highlighted in red and with an arrow indicates the transcription start site (TSS). GATA-like CREs are highlighted inside red boxes. (PDF) [file pgen.1010037.s001.pdf]

S1 Fig

```
P-DBM1Ac-S :TTGGCTGAAC TGATTTTAAT GAATCATGGT TAAACACAAA CAAGACAAGA AAACAAACTA TGATCCAATT CAACGCCAAA CAGAAACAAA :-2207
P-NIL-R    :TTGGCTGAAC TGAGTTTAAT GAATCATGGT TAAACACAAT CAAGACAAGA AACCAAACTT TGATCCAATT CAACGCCAAA CAGATACAAA :-1913
            TTGGCTGAAC TGA TTTAAT GAATCATGGT TAAACACAA  CAAGACAAGA AA CAAACT  TGATCCAATT CAACGCCAAA CAGA ACAAA

P-DBM1Ac-S :CTCATAACAC CCCTATTTTG TAAGATCGGA GTTTTAATAT AATTAAGTAT GTTTATAATT ATCATCATTT TAGTAGTTCG ATAAAACAAA :-2117
P-NIL-R    :CTCATAACAC CCCTATTTTG TAAGATCGGA GTTTTAATAT AATTAAGTAT GTTTATAATT ATCATCATTT TAGTAGTTCG ATAAAACAGA :-1823
            CTCATAACAC CCCTATTTTG TAAGATCGGA GTTTTAATAT AATTAAGTAT GTTTATAATT ATCATCATTT TAGTAGTTCG ATAAAACA A

P-DBM1Ac-S :TCACAGTATT TCAGTAAAAA ACATCATCCT TTACTCTAAC TTAGTTCAGA GATATAAGGC GGGACTAGAC GGCGCTCTCT TGGCAGTACA :-2027
P-NIL-R    :TCACAGTATT TCAGGAAAAA ACATCATCCT TTACTCAAAC TTAGTTCAGA GATATAAGGC GGGACTAGAC GGCGCTCTCT TGGCAGTACA :-1733
            TCACAGTATT TCAG AAAAA ACATCATCCT TTACTC AAC TTAGTTCAGA GATATAAGGC GGGACTAGAC GGCGCTCTCT TGGCAGTACA

P-DBM1Ac-S :GAGACGCGCG CACGCCACGT GCGGTCATTA AGCTTCATTA TTCATTAACT TCATTATCAT TATTTATTAA TTTCATTATA ---------T :-1946
P-NIL-R    :GAGACGCGCG CACGCCACGT GCGGTCATTA AGGTTCATTA TTCATTAACT TCATTATCAT TATTCATTAA TTTCATTATA ATATCATAAT :-1643
            GAGACGCGCG CACGCCACGT GCGGTCATTA AG TTCATTA TTCATTAACT TCATTATCAT TATT ATTAA TTTCATTATA          T

P-DBM1Ac-S :CGGTTAATTC GCTATTTCAT ACGCGATGGC ACTTTTCGCA GTAATCGTCC TTTTGAGCGT CAGCCAAACG TTTGGCGCCA ATTTTTTTGG :-1856
P-NIL-R    :CCGTTAATTC AAGACTTCAT ACGCGATGGC ACTTTTCGCA GTAATCGTCC TTTTGAGCGT CAGCCAAACC TTTGGCGCCA ATTTTTTCGG :-1553
            C GTTAATTC    A TTCAT ACGCGATGGC ACTTTTCGCA GTAATCGTCC TTTTGAGCGT CAGCCAAAC  TTTGGCGCCA ATTTTTT GG

P-DBM1Ac-S :TAAGTTTTCT GTTTTAGGTT TTTATTTAAT TATTCCGATT TCTTAGGTAC GTAGACTCTT ATCGTGTAAA CGCTGAAAAA AATA-AGCGT :-1767
P-NIL-R    :TAAGTTTTA- -----AGGTT TTTATTCTAT TATTCCGATT ACTAAGGTAG TTAGACTGTT ATCGTGTAAA CGCTGAAATA AATTTAGCGT :-1469
            TAAGTTTT        AGGTT TTTATT  AT TATTCCGATT  CT AGGTA   TAGACT TT ATCGTGTAAA CGCTGAAA A AAT  AGCGT

P-DBM1Ac-S :CTACCTATCG GTACGTATGG GTTCACGAAT CCTAACTAAT ATTATAAATG CGAAAGTAAC TGTGTCTGTC TGTCTGTCTG TCTGTCTGTC :-1677
P-NIL-R    :CTACCTATTG GTAT------ ---------- ---------- ---------- ---------- ---------- ---------- ---------- :-1455
            CTACCTAT G GTA

P-DBM1Ac-S :TGTTACTCTT TCACGCTAAA ACTACTGAAC CGATTTGAAT GAAATTTGGT ATACGTAAGG TCTAGACCCT GGGAAAGAAC ATAGGCTACT :-1587
P-NIL-R    :---------- ---------- ---------- ---------- ---------T GTACGTATGG ---------- ---------- ---------- :-1446
                                                             T  TACGTA GG

P-DBM1Ac-S :TTTTATCCAG GAATTCCCAC GGGAAAACTT TTTAATGCGA AGCGAAGCGC GCGGGAACAG CTAGTTTTTC ATAAATGTAT GGCGTCGGCA :-1497
P-NIL-R    :---------- -------CAC CAGACGAATT TTTCAT---- ----AAATGT ATGGAGAAA- ---------- ---------C GGCGTCGGCA :-1401
                              CAC   GA  A TT TTT AT        AA  G    GG  A A                       GGCGTCGGCA

P-DBM1Ac-S :ATGCCGATTA AGTGGACGCA CTTGTGGCAA TTTCTGTATG AAGAATACTG AGAGAGATGC GTCCGTTACG TACGATGCCG AAAGGTGGGC :-1407
P-NIL-R    :ATGCCGATGG AGTGGACGCA CTTGTGAGAA TTTTTGT--- ---------- ---------- ---------- ---------- ---------- :-1364
            ATGCCGAT   AGTGGACGCA CTTGTG  AA TTT TGT

P-DBM1Ac-S :CTTTACAGGT CAGTTTTTTG CAAAAATTCC CGATGAGTCG GTAAGTACTT ----TAAAAA AACCCACAAT ATAATTCGTC ACGCTAAACT :-1321
P-NIL-R    :---------- TAGTTTTTTG CAAAAATTGC CGATGAGTCG GTAGGTACTT ACTTTAAAAA AACCCGCAAT ATAATTCGTT ACGCTGAACT :-1284
                        AGTTTTTTG CAAAAATT C CGATGAGTCG GTA GTACTT     TAAAAA AACCC CAAT ATAATTCGT  ACGCT AACT

P-DBM1Ac-S :TAACACGAAA ATTCATAAAA ATCTTACAGA CATAAGGTGC TAAGTTGAAA GTAGATACCT ACCTATAAGT TTACCAAACA TTGTCTGAAT :-1231
P-NIL-R    :TAACACAAAA AT-CATAAAA ATCTTACAGA CATAAGGTGC TAAGTTGAAA GTAGATACCT ACCTATAAGT TTACCAAACA TTGTCTGAAT :-1195
            TAACAC AAA AT CATAAAA ATCTTACAGA CATAAGGTGC TAAGTTGAAA GTAGATACCT ACCTATAAGT TTACCAAACA TTGTCTGAAT

P-DBM1Ac-S :CTTGCTAGAA TAAGTGGTCC ACCAACCCGC ACTGGACCAT CAACCCGCAC TGGTCCACCA ACCCGCACTA GGCCAGCGTG GTGGATTAGG :-1141
P-NIL-R    :CTTGCTAGAA TAAGTGGTCC ACCAACCCGC ACTGGACCAT CAACCCGCAC TGGTCCACCA ACCCGCACTA GGCCAGCGTG GTGGACTAGG :-1105
            CTTGCTAGAA TAAGTGGTCC ACCAACCCGC ACTGGACCAT CAACCCGCAC TGGTCCACCA ACCCGCACTA GGCCAGCGTG GTGGA TAGG

P-DBM1Ac-S :CCTGAAACTA CTCCTTCATT GGAAGGAGAC CCGTGCCCCA GCAGTGGGGA CTGGGTTGTG ACAGTGAAAT GACTATTAGT CCTCTACCTT :-1051
P-NIL-R    :CCTAAAACTT CTCCTTCATT GGAAGGAGAC CCGTGCCCCA GAAGTGGGGA CT-------- ---------- ---------C CTCCTACCT- :-1043
            CCT AAACT  CTCCTTCATT GGAAGGAGAC CCGTGCCCCA G AGTGGGGA CT                               C  CTACCT

P-DBM1Ac-S :TAGTCTCGCC ACCCGATCTT TAGGTTGAAA ATGAACACCT TTGTCTAATT ATGC---ATG TTTTCGTAAA ATCCCAATAT TTTTTGCAAT :-964
P-NIL-R    :---------- -----ATCTT TTGGTTGAAA ATGAACACCT TTGTCTAAAT ATGCTGCATG TTTTCGTAAA ATCCCAAGAT TTTTTGCAAT :-968
                            ATCTT T GGTTGAAA ATGAACACCT TTGTCTAA T ATGC   ATG TTTTCGTAAA ATCCCAA AT TTTTTGCAAT

P-DBM1Ac-S :AATTCTAATA ACATCAAACC ATGCTA-TGA TCTA------ CATAGAAACA GTCGTATTTT T-ATGTCACT TATTACTACC ATTCATCAAT :-882
P-NIL-R    :AATTCTTATA ATATCAAACC ATGATAATAA TATATACATA CATAGAAACA GTCGTATTTT TCATGTCACT TATTACTATT ATTCATCAAT :-878
            AATTCT ATA A ATCAAACC ATG TA T A T TA       CATAGAAACA GTCGTATTTT T ATGTCACT TATTACTA   ATTCATCAAT

P-DBM1Ac-S :TTTCTGACTG TAGGTACTTT ACTTACTCGT GTTCAGTATT GTAATAATCA TTAGAAAATT TCTTAAAATA CAAAAAGCCG TTATAT--GC :-794
P-NIL-R    :TTTCTGACTG TAGGTACTTT ACATACTCGT GTTCAGTATT GTAATAATCA TTAGCAAATT TCTTAAAATA CAAAAAGCCG TTATATATGC :-788
            TTTCTGACTG TAGGTACTTT AC TACTCGT GTTCAGTATT GTAATAATCA TTAG AAATT TCTTAAAATA CAAAAAGCCG TTATAT  GC

P-DBM1Ac-S :TATCTTGCAT GCTATTTTCG GTTATATCTA TAGTTCTATC TTTATCTTAT ATTCAGTGCT TCTTAATGGT AATTCTAAAT AAGTTGGTTA :-704
P-NIL-R    :TATCCTGCAT GCTATTTTCG GTTATATCTA TAGTTCTATC TTTGTCTTAT ATTCAGTGCT TCTTAATGGT AATTCTAAAT AAGTTGGTTA :-698
            TATC TGCAT GCTATTTTCG GTTATATCTA TAGTTCTATC TTT TCTTAT ATTCAGTGCT TCTTAATGGT AATTCTAAAT AAGTTGGTTA

P-DBM1Ac-S :AAATTTCCAT AGTGATGGCC CATTAGTTCA GTACTGAAAA TAAATGTGGA TTACCGATAC TTGTCTAAAA ATAAACATTG TAATACGAAA :-614
P-NIL-R    :AAATTTCCAT AGTGATGGCC CATTAGTTCA GTACTGAAAA TAAATGTGAA TTACAGATAC TTGTCTAAAA ATAAACATTG TAATACGAAA :-608
            AAATTTCCAT AGTGATGGCC CATTAGTTCA GTACTGAAAA TAAATGTG A TTAC GATAC TTGTCTAAAA ATAAACATTG TAATACGAAA

P-DBM1Ac-S :CGGGGTTACT GATCTCTGAA CAAATTTATA GTTCAAGGAT TTTTTTGTTT TATATTAAAA TTAAAATAAA TCACCAATAA AGATTTTCAG :-524
P-NIL-R    :CGGGGTTACT GATCTCTGAA CAAATTTATA GTTCAAAGAT TTTTTTGGTT ---ATTAAAA TTAAAATAAA TCACCAACAA AGATTTTCAG :-521
            CGGGGTTACT GATCTCTGAA CAAATTTATA GTTCAA GAT TTTTTTG TT    ATTAAAA TTAAAATAAA TCACCAA AA AGATTTTCAG

P-DBM1Ac-S :CAAAAATCGA TCCCTGTTTG GTAATTTTAG AACTTACCAA TAAGCATATT CTAATATTAG GTAAACTAAC TTTAATGACA TAATTATAAG :-434
P-NIL-R    :CAAAAATCGA TCCCTGTTTG GTAATTTTAG AACTTACCAA TAAGCATATT CTAATATTAG CTAAACTAAC TTTAATAACA TAATTATAAG :-431
            CAAAAATCGA TCCCTGTTTG GTAATTTTAG AACTTACCAA TAAGCATATT CTAATATTAG  TAAACTAAC TTTAAT ACA TAATTATAAG

P-DBM1Ac-S :AGATAAGGGT AACTGGTAGG TAGGTAGGTA CTTAAATTAA TTTAATTACT TATTCGAAAA AAAACACGCT TAATAAATAA TCGGAACTAA :-344
P-NIL-R    :AGATAAGGGT AACTGGAAGG TAGGTAGGTA TTTAAATTAA TTTAA----T TATTTGAAAA AAAAAACGCT TAGTAAATAA TCGGAAGTAA :-345
            AGATAAGGGT AACTGG AGG TAGGTAGGTA  TTAAATTAA TTTAA    T TATT GAAAA AAAA ACGCT TA TAAATAA TCGGAA TAA

P-DBM1Ac-S :TTAATGAAAC TTCAGGATCT TTAGAATTTA GCCATTGAGA ACGCATGGTC CAAG-TCCGC GCTGTTCTAT GGGATTTACA TTTAAATGTA :-255
P-NIL-R    :TTTATGAAAC TTCAGGATCT TTAGAATTTA ACCATTGAGA ACGCATGATC CAAGGTTCGC GTTGTTCTAT GGGATTTACA TTTAAATGTA :-255
            TT ATGAAAC TTCAGGATCT TTAGAATTTA  CCATTGAGA ACGCATG TC CAAG T CGC G TGTTCTAT GGGATTTACA TTTAAATGTA

P-DBM1Ac-S :TTGATCAAAA GCGTAGCTGA TGATTTCAGA TTGCCGTCGA ACGATCTGCA GCACATGCAA CGATGTTAGT TCGACATTGG GATTGTCCTT :-165
P-NIL-R    :TTGATCAAAA GCGTAGCTGA TGATTTCAGA TTGCCGTCGA ACGATCTGCA GCACATGCAA CGATGTTTGT TCGACATTGG GATTGTCCTT :-165
            TTGATCAAAA GCGTAGCTGA TGATTTCAGA TTGCCGTCGA ACGATCTGCA GCACATGCAA CGATGTT GT TCGACATTGG GATTGTCCTT

P-DBM1Ac-S :TAAAAACTTC ATTTAAACGT TTTGTTTAAC AGCATTTTTA ACCACAATAC ATATGTTAAT CTTTGTCAAT ATCAGCCTAT CGCAGTTGCA :-75
P-NIL-R    :TAAAAACTTC ATTTAAACGT TTTGTTTAAC AGCATTTTTA ACCACAATAC ATATGTTAAT CTTTGTCAAT ATCAGCCTAT CGCAGTTGCA :-75
            TAAAAACTTC ATTTAAACGT TTTGTTTAAC AGCATTTTTA ACCACAATAC ATATGTTAAT CTTTGTCAAT ATCAGCCTAT CGCAGTTGCA

                                                                                             +1
P-DBM1Ac-S :AGTTGCGTCG TGTAGTAATT CATCAATGAG TCAATAATAA TAAAACGCCT TTCCTCGGCG TCTGCGAGTC AGTCATG :+3
P-NIL-R    :AGTTGCGTCG TGTAGTAATT CATCAATGAG TCAATAATAA TAAAACGTCT TTCCTCGGCG TCTGCGAGTC AGTCATG :+3
            AGTTGCGTCG TGTAGTAATT CATCAATGAG TCAATAATAA TAAAACG CT TTCCTCGGCG TCTGCGAGTC AGTCATG
```
