# Supplementary material for: MAPK-mediated transcription factor GATAd contributes to Cry1Ac resistance in diamondback moth by reducing PxmALP expression
Source: PLoS Genet. 2022 Feb 3;18(2):e1010037. doi: 10.1371/journal.pgen.1010037 (PMC8846524; doi:10.1371/journal.pgen.1010037)
Supplement: S2 Fig — The conserved GATA-type zinc finger (Znf) domain of GATAd proteins is highlighted by a red background. PxGATAd (Plutella xylostella, MZ712004), BmGATAd (Bombyx mori, XP_012546211.1), TcGATAd (Tribolium castaneum, EFA09251.2), AaGATAd (Aedes aegypti, EAT41982.1), HsapGATAd (Homo sapiens, NP_001295023.1), PhcGATAd (Pediculus humanus corporis, XP_002428265.1), DmGATAd (Drosophila melanogaster, NP_001260326.1). (PDF) [file pgen.1010037.s002.pdf]

S2 Fig

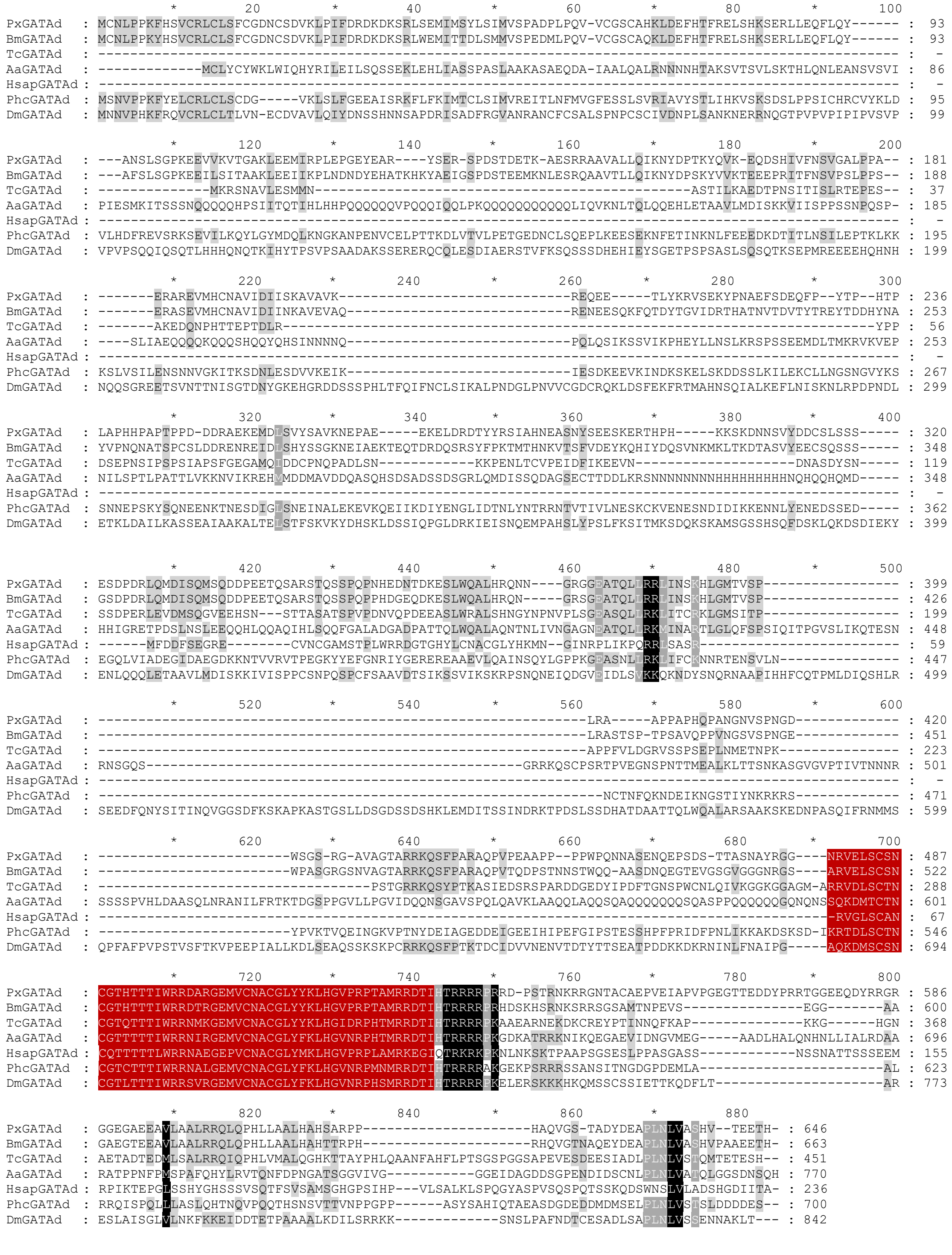
PxGATAd
BmGATAd
TcGATAd
AaGATAd
HsapGATAd
PhcGATAd
DmGATAd
