# Supplementary figures and images for: MAPK-mediated transcription factor GATAd contributes to Cry1Ac resistance in diamondback moth by reducing PxmALP expression
Source: PLoS Genet. 2022 Feb 3;18(2):e1010037. doi: 10.1371/journal.pgen.1010037 (PMC8846524; doi:10.1371/journal.pgen.1010037)

S3 Fig

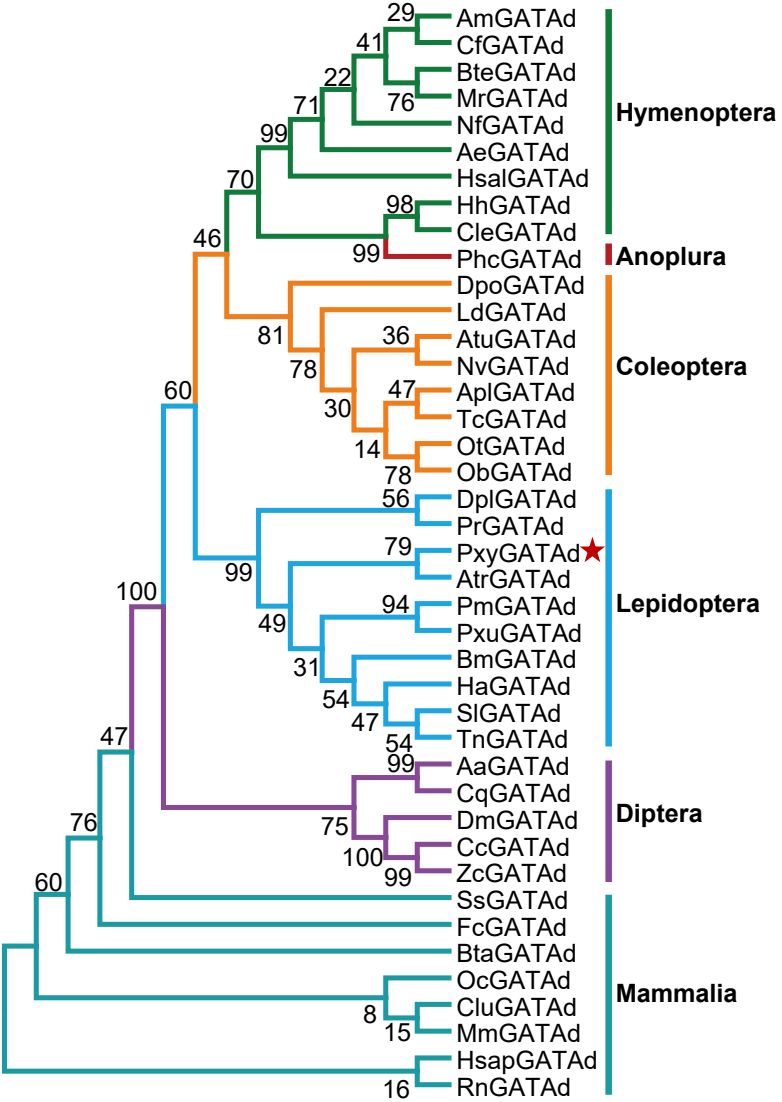

Supplement: S3 Fig — Phylogenetic analysis of the GATAd genes was performed by maximum likelihood method. The GATAd encoding sequence was corrected using NCBI database and transcriptome data of P. xylostella and then cloned from fourth-instar larvae. PxGATAd is indicated by a red star. Full-length amino acid sequences of GATAd genes were retrieved from the GenBank database. Abbreviation: 1. Hymenoptera [Am (Apis mellifera, XP_001120276.2); Cf (Camponotus floridanus, XP_025270700.1); Bte (Bombus terrestris, XP_003400693.1); Mr (Megachile rotundata, XP_003708269.1); Nf (Nylanderia fulva, XP_029159148.1); Ae (Acromyrmex echinatior, XP_011057942.1); Hsal (Harpegnathos saltator, XP_011148075.1); Hh (Halyomorpha halys, XP_014279044.1); Cl (Cimex lectularius, XP_014261884.1)]; 2. Anoplura [Phc (Pediculus humanus corporis, XP_002428265.1]; 3. Coleoptera [Dpo (Dendroctonus ponderosae, XP_019764087.1); Ld (Leptinotarsa decemlineata, XP_023023616.1); Atu (Aethina tumida, XP_019881566.1); Nv (Nicrophorus vespilloides, XP_017769919.1); Apl (Agrilus planipennis, XP_018334804.1); Tc (Tribolium castaneum, EFA09251.2); Ot (Onthophagus taurus, XP_022900328.1); Ob (Oryctes borbonicus, KRT80118.1)]; 4. Lepidoptera [Dpl (Danaus plexippus, OWR52456.1); Pr (Pieris rapae, XP_022123638.1); Pxy (Plutella xylostella, MZ712004); Atr (Amyelois transitella, XP_013199688.1); Pm (Papilio machaon, XP_014367479.1); Pxu (Papilio xuthus, XP_013169273.1); Bm (Bombyx mori, XP_012546211.1); Ha (Helicoverpa armigera, XP_021183187.1); Sl (Spodoptera litura, XP_022834873.1); Tn (Trichoplusia ni, XP_026725121.1)]; 5. Diptera [Aa (Aedes aegypti, EAT41982.1); Cq (Culex quinquefasciatus, EDS42992.1); Dm (Drosophila melanogaster, NP_001260326.1); Cc (Ceratitis capitata, XP_004537903.1); Zc (Zeugodacus cucurbitae, XP_011186611.2)]; 6. Mammalia [Ss (Sus scrofa, NP_999458.1); Fc (Felis catus, XP_011279866.2); Bta (Bos taurus, CAC69835.1); Oc (Oryctolagus cuniculus, XP_008247101.1); Clu (Canis lupus, XP_025318689.1); Mm (Mus musculus, B [file pgen.1010037.s003.pdf]

**S4 Fig**

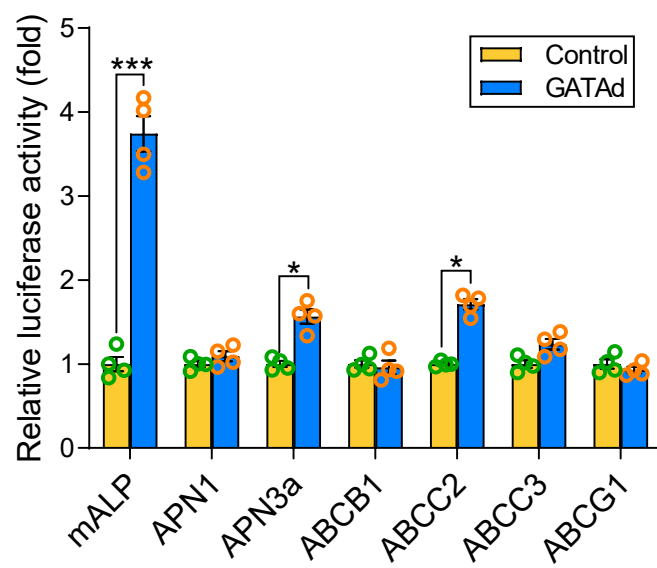

Supplement: S4 Fig — The data are represented by the means and the corresponding SEM values. Four independent transfections were conducted for each pair of plasmids. Holm-Sidak’s test was used for statistical analysis (*p < 0.05, ***p < 0.001). (PDF) [file pgen.1010037.s004.pdf]
