# Supplementary material for: MAPK-mediated transcription factor GATAd contributes to Cry1Ac resistance in diamondback moth by reducing PxmALP expression
Source: PLoS Genet. 2022 Feb 3;18(2):e1010037. doi: 10.1371/journal.pgen.1010037 (PMC8846524; doi:10.1371/journal.pgen.1010037)
Supplement: S1 Table — (PDF) [file pgen.1010037.s005.pdf]

S1 Table. Sequence of the primers used for cloning the promoter region of receptor genes and construction of pGL4.10 recombinant plasmids.

Purpose	Gene	Primer name	Primer sequence (5′-3′)	PCR product size (bp)
Promoter cloning	mALP	PxmALP-F	TTGGCTGAACTGATTTTAATG	2393/2101
		PxmALP-R	CCTTCTTGGGCTTCGGTGT	
	APN1	PxAPN1-F	AGCTTCCTCTCAGTGTTCG	1785
		PxAPN1-R	TCTGTTTGTAGGTTGCCCC	
	APN3a	PxAPN3a-F	GCAGTATGGGCAGTAAGAA	2339
		PxAPN3a-R	CATCGGTGAAAATGGTGTT	
	ABCB1	PxABCB1-F	TTAGTTACATAGTTACCGACAC	1272
		PxABCB1-R	TTGAGTCTTCGTTGAAATT	
	ABCC2	PxABCC2-F	ATTTCCCGATAGGCTGTGA	1480
		PxABCC2-R	GGCTTGCCCTTCTTCACCT	
	ABCC3	PxABCC3-F	AATACCTCACCTGCCCACC	2148
		PxABCC3-R	CATCCTCCGCAACCTTCAC	
	ABCG1	PxABCG1-F	ATGTCGATGTCGGTGGTT	2326
		PxABCG1-R	ATCAGAGGCTCCTGCTCC	

PGL4.10 recombinant	mALP	†P(-2296/-1)-F	TTGGCTGAACTGATTTTAATG AATC	2296/2004
		†P(-1921/-1)-F	TCGGTTAATTCGCTATTTCATACGC	1921/1620
		†P(-1753/-1)-F	CGTATGGGTTCACGAATCCTAAC	1753/1458
		†P(-1395/-1)-F	AGTTTTTTGCAAAAATTCCCGATG	1395/1053
		†P(-1125/-1)-F	TCATTGGAAGGAGACCCGTG	1125/1089
		†P(-577/-1)-F	GGATTTTTTTGTTTTATATTAAAAT	577/571
		†P(-388/-1)-F	TTACTTATTCGAAAAAAAACACG	388/385
		†P(-218/-1)-F	TCGAACGATCTGCAGCACATGC	218/218
		‡Promoter-R	GACTGACTCGCAGACGCC	—
	APN1	†P(-1216/-1)-F	AGCTTCCTCTCAGTGTTCAC	1216
		‡Promoter-R	TTTCTCGCCAGTATAAAGCTA	
	APN3a	†P(-2233/-1)-F	GCAGTATGGGCAGTAAGAA	2233
		‡Promoter-R	TTCATTACTTATCTAATTTATTTTAG	
	ABCB1	†P(-1247/-1)-F	TTAGTTACATAGTTACCGACACAC	1247
		‡Promoter-R	TTTGACTGTACGTTTTTATATCTT	
	ABCC2	†P(-1364/-1)-F	ATTTCCCGATAGGCTGTGAA	1364
		‡Promoter-R	TTCGACACATGTAGTTTGCAC	
		†P(-2123/-1)-F	AATACCTCACCTGCCCACC	

ABCC3	‡Promoter-R	TCTTATATCACTATTAATTAATTAAT	2123
ABCG1	†P(-2291/-1)-F	ATGTCGATGTCGGTGGTTTAGC	2291
	‡Promoter-R	TTTGTAAAAAAAAAACACTAATCG	

Restriction enzymes KpnI and BglII were used to cut the pGL4.10 reporter vector and generate a linearized vector, PCR primers used to amplify the various length of promoters contained 15 bp extensions homologous to the cut vector ends.

†A 15 bp sequence (TGGCCTAACTGGCCG) is added to the 5′-ends of the forward (F) primers for construction of pGL4.10 recombinants containing gene promoters.

‡A 15 bp sequence (CGCCGAGGCCAGATC) is added to the 5′-ends of the reverse (R) primer for construction of pGL4.10 recombinants containing gene promoters.
