# Supplementary material for: MAPK-mediated transcription factor GATAd contributes to Cry1Ac resistance in diamondback moth by reducing PxmALP expression
Source: PLoS Genet. 2022 Feb 3;18(2):e1010037. doi: 10.1371/journal.pgen.1010037 (PMC8846524; doi:10.1371/journal.pgen.1010037)
Supplement: S2 Table — (PDF) [file pgen.1010037.s006.pdf]

S2 Table Sequence of the primers used for site-directed mutagenesis

Primer name	Primer sequence (5′-3′)	PCR product size (bp)
CRE1-Mu1-F	ACTGAAAATAAATGTGAATTACAGATACTTGTCT	1125
CRE1-Mu1-R	AATGTTTATTTTTAGACAAGTATCTGTAATTCAC	
CRE1-Mu2-F	ACTGAAAATAAATGTGAACGACATGCGCTTGTCT	1125
CRE1-Mu2-R	AATGTTTATTTTTAGACAAGCGCATGTCGTTCAC	
CRE5-Mu1-F	TATCAAACCATGCTATGATATATACATA	1088
CRE5-Mu1-R	CTGTTTCTATGTATGTATATATCATAGCAT	
CRE5-Mu2-F	TATCAAACCATTCGCCGAATATATACATA	1089
CRE5-Mu2-R	ACTGTTTCTATGTATGTATATATTCGGCGAAT	
CRE5-Mu3-F	ATATCAAACCATGATAATAATCTATACATA	1089
CRE5-Mu3-R	CGACTGTTTCTATGTATGTATAGATTATT	
CRE5-Mu4-F	TATCAAACCATGATAATAATATACATAG	1083
CRE5-Mu4-R	ATACGACTGTTTCTATGTATATTATTATC	
CRE5-Mu3/4-F	TATCAAACCATGATAATAATCTACATAG	1083
CRE5-Mu3/4-R	ATACGACTGTTTCTATGTAGATTATTATC	
