# Supplementary material for: MAPK-mediated transcription factor GATAd contributes to Cry1Ac resistance in diamondback moth by reducing PxmALP expression
Source: PLoS Genet. 2022 Feb 3;18(2):e1010037. doi: 10.1371/journal.pgen.1010037 (PMC8846524; doi:10.1371/journal.pgen.1010037)
Supplement: S3 Table — (PDF) [file pgen.1010037.s007.pdf]

S3 Table Sequence of the primers used for cloning TFs from *P. xylostella*

Primer name	Primer sequence (5′-3′)	PCR product size (bp)
GATAa-F	TCTCGTTGGCTCTGAGCATG	1139
GATAa-R	GGCTTATTATCGCTGCGTGC	
GATAb-F	TGATCTAGATATGAGTGTGTGG	1823
GATAb-R	TAAGAAGACTGGACAGCGGAG	
GATAc-F	GGCCAGTGACTTTTAGTGTTG	1545
GATAc-R	AGGTCGGGAGTGTCGTGT	
GATAd-F	CGTTTGGTTGACAATCGCGA	2176
GATAd-R	TGGAACTGTCAAAGGAGGCC	
GATAe-F	CACGAGCAGCAGCATCTAGA	1845
GATAe-R	TCAGAGCGCATATCTACAGA	
Dfd-F	TGACGGCGGCCATGTTAGCAG	1213
Dfd-R	AACATTGTTTAAAGATTATAAGG	
Antp-F	TTAGTGTGGCCCCCAGGCCCG	972
Antp-R	TCCTAAGCTAGAGTTATTGTG	
Hb-F	AGTTTCAGAACATTAGTGCG	

Hb-R	CTAGACCACCCGCACGTTAGACCA	1945
Ftz-F	GTGAAAGTGAAGTGAGACCAA	1442
Ftz-R	CATTACGTGAATGCCCATCACAT	
POU6F2-F	TCAACAGACGAGTCGGGATGCG	1891
POU6F2-R	GATAAACTAAACCCACCCAT	
FoxA-F	CCGAGTGGTAGTGTTTGCG	1095
FoxA-R	CCAGCGGGTACTGTCACAAG	
