# Supplementary material for: MAPK-mediated transcription factor GATAd contributes to Cry1Ac resistance in diamondback moth by reducing PxmALP expression
Source: PLoS Genet. 2022 Feb 3;18(2):e1010037. doi: 10.1371/journal.pgen.1010037 (PMC8846524; doi:10.1371/journal.pgen.1010037)
Supplement: S4 Table — (PDF) [file pgen.1010037.s008.pdf]

S4 Table Primers used for constructing the recombinant plasmids of TFs

Primer name	Primer sequence (5′-3′)	PCR product size (bp)
[§]In-GATAa-F	ATGTTTAGTCTGCAGCAGGTG	954
[‡]In-GATAa-R	CGTGCTCGCCATGAGCTTAG	
[§]In-GATAb-F	ATGGAGGCCGGCGCGCAC	1656
[‡]In-GATAb-R	GCTGCCCAGCGACACGAC	
[§]In-GATAc-F	ATGGTTTGTGAGGCCGAAGC	1434
[‡]In-GATAc-R	CGTGTACTCACTTCTCCAGC	
[§]In-GATAd-F	ATGTGTAATTTGCCGCCGAAG	1938
[‡]In-GATAd-R	GTGCGTCTCCTCCGTGACGT	
[§]In-GATAe-F	ATGGAAGGCGAAGGTCACGAG	1686
[‡]In-GATAe-R	CCCGCGCCACTCACCGCCCA	
[§]In-Dfd-F	ATGCGCGACCCCGCCCCG	1155
[‡]In-Dfd-R	TAAGGCGGTGAGACCGTAGTCTG	
[§]In-Antp-F	ATGAGCGCCAACAACTGC	885
[‡]In-Antp-R	TTGTGGCGAGGTGGGCGG	
[§]In-Hb-F	ATGCTGAGCTGCGCGCCTGC	

[‡]In-Hb-R	GTTGTGCTGCGCGCGACC	1863
[§]In-POU6F2-F	ATGTCGGAGGGCGCGGAGGGGGC	1776
[‡]In-POU6F2-R	GACCATGCCTTTTGACATCATTC	
[§]In-Ftz-F	ATGTCATCCGTGGCTACTAC	1404
[‡]In-Ftz-R	CATTTTAGGTACCATTCCTCCGT	
[§]In-FoxA-F	ATGATCTCGCAGAAGCTGTCGTAC	1029
[‡]In-FoxA-R	CAAGGGCGGCTGCGCGTG	
[¶]GATAd-Y1H-F	ATGTGTAATTTGCCGCCGAAG	1938
[*]GATAd-Y1H-R	GTGCGTCTCCTCCGTGACGT	

Restriction enzymes KpnI and XhoI were used to cut the pAc5.1/V5-His B reporter vector and generate a linearized vector, PCR primers used to amplify the various length of promoters contained 15 bp extensions homologous to the cut vector ends.

[§]A 15 bp sequence (AGACCCCGGATCGGG) is added to 5′-ends of forward primers of pAc5.1-GATAs recombinant plasmids.

[‡]A 15 bp sequence (GCCCTCTAGACTCGA) is add to 5′-ends of reverse primers of pAc5.1-GATAs recombinant plasmids.

[¶]A 15 bp sequence (GGAGGCCAGTGAATT) is added to the 5′-end of forward primer of pGADT7-GATAd recombinant plasmid.

[*]A 15 bp sequence (TCATCTGCAGCTCGA) is added to the 5′-end of the reverse primer of pGADT7-GATAd recombinant plasmid.
