# Supplementary material for: MAPK-mediated transcription factor GATAd contributes to Cry1Ac resistance in diamondback moth by reducing PxmALP expression
Source: PLoS Genet. 2022 Feb 3;18(2):e1010037. doi: 10.1371/journal.pgen.1010037 (PMC8846524; doi:10.1371/journal.pgen.1010037)
Supplement: S5 Table — (PDF) [file pgen.1010037.s009.pdf]

S5 Table Sequence of the primers used for real time qPCR analyzes and dsRNA synthesis

Primer name	Primer sequence (5′-3′)	PCR product size (bp)
qGATAd-F	CCCGACCGGCTACAGAT	112
qGATAa-R	CGGTGTTGTCTTCGTGGTT	
qGATAe-F	AAGGTCACGAGCACATGGAG	207
qGATAe-R	GGTGTTGTTGCTGCTCGATG	
qPOU6F2-F	CACTGAAGGGCCAGCTTACA	288
qPOU6F2-R	TGCGCGTTCAACAGTTCAAG	
qMAP4K4-F	CATCAACTGGCTCCGTCTG	180
qMAP4K4-R	TCATCTTCGGTGACATCCATC	
qALP-F	GCACACACCATGACCGTAGCAG	169
qALP-R	GGCTCTTCGTGACATCG	
qRPL32-F	CCAATTTACCGCCCTACC	120
qRPL32-R	TACCCTGTTGTCAATACCTCT	
dsGATAd-F†	T7-AGAGCCATCGGACTCTACGA	329
dsGATAd-R†	T7-GTGGGTAGTCGTCCTCGGTA	
dsMAP4K4-F†	T7-GCCCGAGATACGCAAATACA	

dsMAP4K4-R[†]	T7-CCGAGCCATAGATCACTTTCA	582
dsEGFP-F[†]	T7-CCACAAGTTCAGCGTGTCCG	469
dsEGFP-R[†]	T7-AAGTTCACCTTGATGCCGTTC	

[†]Forward and reverse primers used to synthesize specific dsRNA templates contained the T7 RNA polymerase promoter sequence (5′-TAATACGACTCACTATAGGGAGA-3′) appended to both their 5′- and 3′-ends.
