# Supplementary material for: MAPK-mediated transcription factor GATAd contributes to Cry1Ac resistance in diamondback moth by reducing PxmALP expression
Source: PLoS Genet. 2022 Feb 3;18(2):e1010037. doi: 10.1371/journal.pgen.1010037 (PMC8846524; doi:10.1371/journal.pgen.1010037)
Supplement: Attachment — Submitted filename: Response to Reviewers.docx [file pgen.1010037.s011.docx]

**Response to Reviewers’ comments**

Subba Reddy Palli, Associate Editor

Gregory P. Copenhaver, Editor-in-Chief

*PLoS Genetics*

January 2, 2022

Dear Editors,

We would like to thank you, and the three anonymous expert reviewers, for the time spent reviewing our manuscript (Manuscript Number: PGENETICS-D-21-01514) entitled “MAPK-mediated transcription factor GATAd contributes to Cry1Ac resistance in diamondback moth by reducing *PxmALP* expression” submitted to *PLoS Genetics*. The insightful and constructive comments from the three reviewers have been instrumental in improving the manuscript. We have incorporated their suggested changes in the revised manuscript and highlighted them in yellow. We hope you will find the new version of the manuscript suitable for publication.

The following is a point-by-point response to the reviewer’s comments:

**Responses to Reviewers’ Questions**

**Reviewer #1**: In this manuscript the genetic mechanisms behind down-regulation of *PxmALP* in a Cry1Ac resistant strain of *Plutella xylostella* are characterized. Both a transcription factor and a *cis*-acting sequence are identified as participating in down-regulation of *PxmALP* in the resistant insects. The data presented advances our understanding of the genetic control of Cry toxin receptors and how insects may down-regulated their expression to reduce susceptibility to these toxins.

-The manuscript is well written, although I consider the choice of references is not appropriate in some instances:

**Response:** We thank reviewer-1 for recognizing the relevance of our data and their positive comments, and we hope that our responses and actions described below will address the raised concerns.

1. Line 73: This sentence is about evolution of resistance to pesticides worldwide, yet reference 1 is too specific to Drosophila melanogaster, which is not a relevant pest of crops. A reference to a review on insect resistance to pesticides would be more appropriate.

**Response:** We have substituted the inappropriate reference with a more comprehensive reference based on the suggestion of reviewer.

2. Line 75: This statement is on Bt as alternative to synthetic pesticides, yet reference 2 refers to mechanisms of resistance to Bt and does not provide any details on comparisons to synthetic pesticides. A review on the use and benefits of Bt versus chemical pesticides would be more appropriate.

**Response:** Since the above reference change covers both chemical and biopesticides we have modified this reference, and the associated text, to just provide background information on Bt.

3. Line 86: Reference 12 is the same as reference 3 and is focused on mechanisms of resistance, not on describing Bt midgut receptors. Reference 35 would be more appropriate here.

**Response:** We apologize for the duplication of reference 2/12. We have changed the text to specific references that describe receptors (ref 12) and their role in resistance (ref 13).

4. Line 88: Reference 12 (or 3) would be more appropriate here as it presents a review of cases in which reduced expression of receptors are involved in resistance to Bt toxins.

**Response:** Changed as described above

5. Line 122: Reference 12 does not provide any data supporting the physiological role of ALPs. Probably no reference is needed here as this is well established.

**Response:** We have taken the advice of the reviewer and removed this reference.

6. Fig. 2 shows that GATAd expression is 50% reduced in resistant compared to susceptible larvae, yet in Fig. 7 it is shown that this same level of knock down results in a slight difference (albeit significant) in susceptibility. The authors should discuss this observation and provide explanations for not observing a more relevant resistance phenotype in Fig. 7B (perhaps reduced expression of other receptors does not occur in RNAi experiments contrary to data with cell cultures in Fig. S4?).

**Response:** We do indeed believe that the other receptors are not primarily under the control of GATAd and so the small change in susceptibility upon GATAd knock down is just due to the loss of one of the receptors (ALP). We have clarified this viewpoint in lines 288-292 and 322-326.

7. It is interesting that the manuscript presents basically two mechanisms (one trans and one cis) to reduce PxmALP expression. How would two mechanisms resulting in the same phenotype be selected in the same strain? This needs to be discussed.

**Response:** We believe that *trans* mechanisms are the dominant ones that control the response of *P. xylostella* to Cry1Ac and which are constitutively altered in the resistant populations. In a resistant population there is then less selective pressure to retain normal levels of receptor expression and so additional mutations can accumulate and even be selected for if they enhance the resistance phenotype. We have discussed this in lines 390-396.

**Reviewer #2**: The manuscript entitled ‘MAPK-mediated transcription factor GATAd contributes to Cry1Ac resistance in diamondback moth by reducing *PxmALP* expression’ deciphered that the expression of *PxmALP* was *cis*-regulated by transcription factor GATAd, and lay a solid foundation for comprehensive understanding the mechanisms of Cry1Ac resistance in diamondback moth. The experiments were well designed and scientifically sound, and the manuscript was well written. I recommend the acceptance of the manuscript.

**Response:** We thank reviewer-2 for recognizing the relevance of the presented data and for their positive comments.

**Reviewer #3**: Alkaline phosphatase (ALP) is one of the midgut receptors of Bt toxins. This paper identified that *PxGATAd* directly activates *PxmALP* expression via interacting with a non-canonical but specific GATA-like cis-response element (CRE) located in the *PxmALP* promoter region. In addition, *PxGATAd* is trans-regulated by the upstream activated MAPK signaling pathway. The manuscript was well organized and written. The data are sufficient to support the conclusion in the manuscript.

**Response:** We thank the reviewer for the precise summary of our work, and we hope the responses described below can address the raised concerns by the reviewer.

There are two minor points:

1. lines 339-340, HamALP was mentioned. Are there any reports on the TFs of mALP in other insects? If so, please discuss more about the TFs of mALP in insects.

**Response:** As mentioned in lines 339-341 GATAe was demonstrated to be a transcription factor of HaALP in insect cell lines. It has been reported that the forkhead transcription factor FKHR stimulates ALP promoter activity through the FRE (forkhead response element) in its promoter in humans (Hatta M, Daitoku H, Matsuzaki H, Deyama Y, Yoshimura Y, Suzuki K, et al. Regulation of alkaline phosphatase promoter activity by forkhead transcription factor FKHR. Int J Mol Med. 2002;9(2):147–152) but little else has been reported concerning the control of expression of ALP.

2. There are a few isoforms of GATA. Here, authors found that GATAd was most important. How about their functions in other related studies.

**Response:** We have provided some background information on other isoforms of GATA in lines 330-342 including the role of GATAe in ALP expression in a different insect. We are not aware of other studies that are directly relevant to our work.

We have carefully revised the manuscript according to the reviewers’ suggestions. Hopefully, our efforts and the revised manuscript can ease reviewers’ concerns and meet the journal standards. Please contact me if further modifications are required.

Sincerely Yours

Youjun Zhang
